# Associations of *FKBP5* polymorphisms and methylation and parenting style with depressive symptoms among Chinese adolescents

**DOI:** 10.1186/s12888-021-03576-6

**Published:** 2021-11-09

**Authors:** Lan Guo, Wanxin Wang, Yangfeng Guo, Xueying Du, Guangduoji Shi, Ciyong Lu

**Affiliations:** 1 Department of Medical Statistics and Epidemiology, School of Public Health, Sun Yat-sen University, 74 Zhongshan Rd 2, Guangzhou, 510080 People’s Republic of China; 2 Guangdong Engineering Technology Research Center of Nutrition Translation, Guangzhou, 510080 People’s Republic of China; 3 Health Promotion Centre for Primary and Secondary Schools of Guangzhou Municipality, Guangzhou, 510080 China

**Keywords:** *FKBP5* polymorphism and methylation, Parenting style, Depressive symptoms, Chinese adolescents, Nested case-control study

## Abstract

**Background:**

Genetic factors may interplay with environmental stressors to contribute to risks of depressive symptoms. This study aimed to investigate the association of *FKBP5* polymorphisms and DNA methylation with depressive symptoms among Chinese adolescents, considering the role of parenting style.

**Methods:**

This study used a nested case-control study design based on a cohort study, and the case (*n* = 120) and control groups (*n* = 118) were matched with age. Depressive symptoms, parenting style, and other demographics were measured. Fourteen potential polymorphisms and one promoter region in the *FKBP5* gene were selected for genotyping and methylation analysis.

**Results:**

In the adjusted models, a significant association between *FKBP5* rs7757037 and depressive symptoms was found in the codominant model (AG vs. GG; adjusted odds ratio [AOR] = 2.56, 95% CI = 1.13–5.78) and dominant model (AA+AG vs. GG; AOR = 2.38, 95% CI = 1.11–5.120); rs2817032 and rs2817035 polymorphisms were associated with depressive symptoms in the codominant model and dominant model. Significant interactions between rs7757037 and the father’s parenting style were found in the codominant model (*P* = 0.043) and dominant model (*P* = 0.043), but the gene-environment interactions were not significant after correcting for multiple testing. Moreover, the significant main effects of *FKBP5* methylation status on depressive symptoms were not observed, and there was no significant interaction between *FKBP5* methylation status and parenting style on depressive symptoms.

**Conclusions:**

Further studies are required to confirm the effect of *FKBP5* polymorphisms and methylation as well as their interactions with parenting styles in larger samples.

**Supplementary Information:**

The online version contains supplementary material available at 10.1186/s12888-021-03576-6.

## Background

Depressive symptoms are one of the major mental health problems worldwide and are the main contributor to the global burden of disease in young people [1]. Adolescence represents a developmental transition period between childhood and adulthood, characterized by marked changes in biological systems and physical maturation of the body and brain, rendering adolescents vulnerable to mental health problems, including depressive symptoms [2]. However, the onset of depressive symptoms in adolescence has long-lasting effects on the adolescents’ physical and brain development and may be a significant risk factor for clinical depression later, leading to serious social and educational impairments [3]. However, the pathological mechanism of depressive symptoms has not been adequately studied.

Evidence suggests that the dysregulated and dysfunctional stress response system (i.e., hypothalamic-pituitary-adrenal (HPA) axis activity and glucocorticoid receptor (GR) sensitivity) has been one of the potential biological mechanisms of depression [4]. Meanwhile, the FK506 binding protein 51 (FKBP51) is the co-chaperone of heat shock protein (Hsp) 90 and GR, which can inhibit GR sensitivity to regulate the HPA axis and is highly expressed after stress exposure [5]. Then, the *FKBP5* gene locating on chromosome 6p21.31 (GRCh38), which encodes FKBP51 protein, is an essential regulatory in the HPA stress regulation system [6].

Since the *FKBP5* gene may be involved in the process of depressive symptoms development, a previous animal study has found a relevant role of the *FKBP5* gene in modulating GR sensitivity and enhancing negative glucocorticoid feedback within the HPA axis through mice model lacking the *FKBP5* gene (51KO mice) [7]. Human genetic studies have also reported significant associations between *FKBP5* variants and depressive symptoms [8–11], although the results are inconsistent [12, 13]. For instance, Shimasaki et al. reported a positive association between rs1360780 and depressive state [14]; Scheuer et al. did not find any significant associations of the five *FKBP5* single-nucleotide polymorphisms (SNPs) with the risk of depression, including rs1360780, rs3800373, rs9296158, rs9470080, and rs4713916) [15].

Moreover, besides genetics, DNA methylation is an epigenetic modification that regulates gene expression without changing the DNA sequence [16]. DNA methylation changes can affect gene expression related to the stress regulation system, which has been reported to play a vital role in the pathogenesis of mental disorders [17]. Prior evidence has shown that alteration in DNA methylation of the *FKBP5* gene may be associated with mental disorders such as depression or depressive symptoms [18], which remains to be studied.

It has been widely reported that both genetic factors and environmental stressors play a role in the pathogenesis of depressive symptoms. A recent study showed the heritability of 40% for depression in a young adult cohort [19], suggesting genetic factors may interplay with environmental stressors to contribute to risks of depressive symptoms. Previous evidence suggests that the interactions between environmental stressors and *FKBP5* rs3800373/ rs9296158/ rs1360780/ rs9470080 were statistically significant in a sample of clinically depressed adolescents [13], and the interaction effects of childhood physical abuse and *FKBP5* rs3800373/ rs1360780/ rs4713916 on depressive symptoms in Chinese adolescents were significant [20]. There is evidence that DNA methylation of the *FKBP5* gene is modified by external environmental factors [18]. Furthermore, it has been suggested that childhood trauma exposure interacts with the *FKBP5* T allele could lead to reduced methylation in intron 7 of the *FKBP5* gene, which may affect depressive symptoms [21].

Although adolescents may experience many environmental stressors, parenting style (referring to general patterns of parental behavior) is critically important for adolescent health [22], especially in a developmental period characterized by a rapid elevation in depressive symptoms. Based on the Baumrind’s theory, negative parenting style (e.g., authoritarian or neglectful parenting style) can be categorized as a significant environmental stressor. Previous evidence also suggested that authoritarian and neglectful parenting styles were associated with higher depressive symptoms in adolescents [23, 24]. However, few studies considered the interaction effects between parenting style and *FKBP5* SNPs on depressive symptoms, particularly among adolescents. Scarce studies examined the role of parenting style in influencing *FKBP5* DNA methylation and depressive symptoms in adolescents. Therefore, the aims of this nested case-control study among Chinese adolescents were twofold, to investigate the association of *FKBP5* genetic and epigenetic variation with depressive symptoms among Chinese adolescents and to investigate the potential role of parenting style on these associations.

## Methods

### Study design and participants

**Fig. 1:**
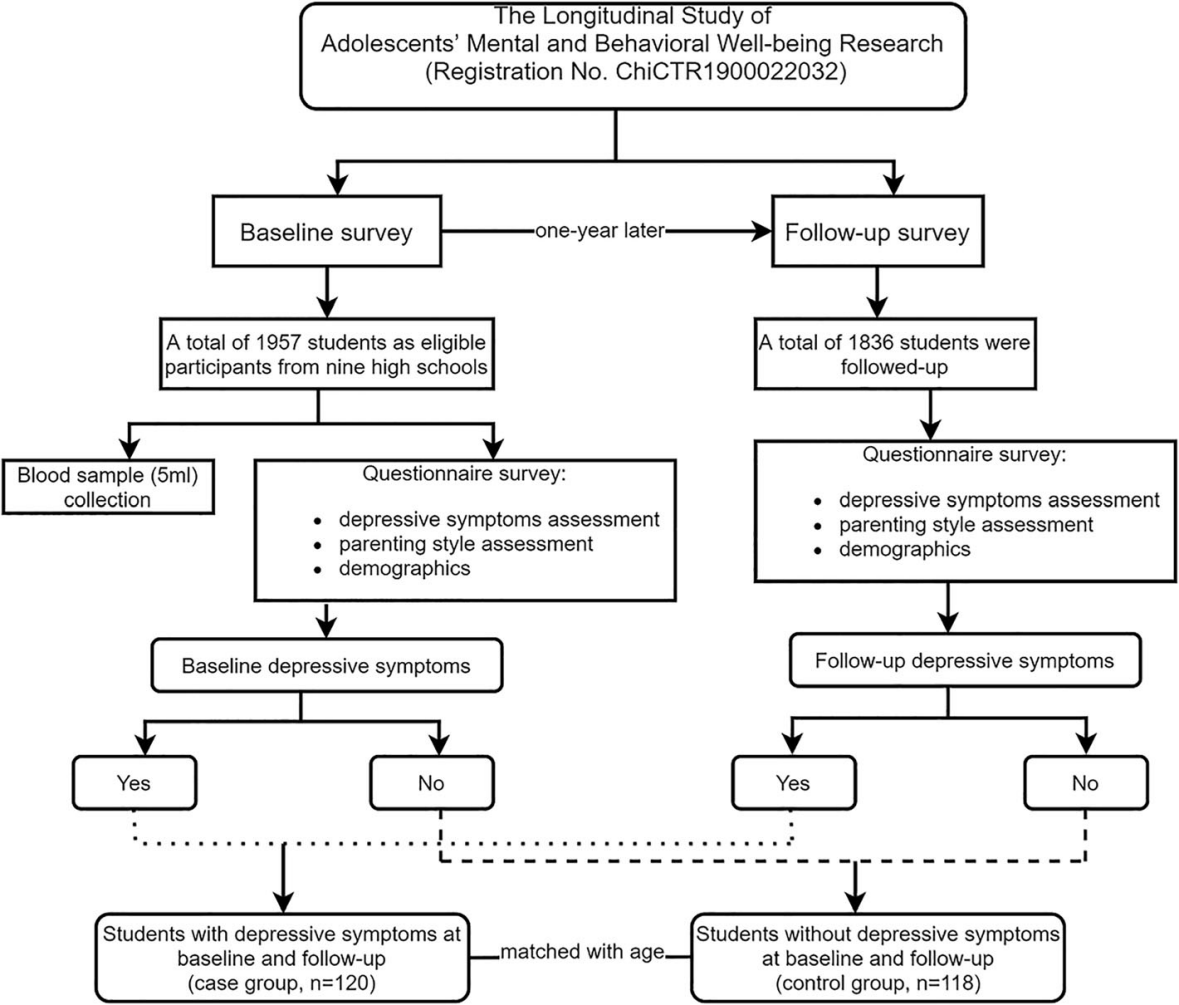
The flowchart of the nested case-control study

We used a nested case-control study design based on the Longitudinal Study of Adolescents’ Mental and Behavioral well-being Research (LSAMBR) in Guangzhou, China (Registration No. ChiCTR1900022032). The LSAMBR is a prospective follow-up study, which adopted a multi-stage, stratified cluster, random sampling method to include 1957 students as eligible participants from nine high schools at baseline (response rate: 99.03%) and followed up one year later (retention rate: 93.8%) [25, 26]. Inclusion criteria of the LSAMBR were providing written informed consent; exclusion criteria included: 1) diagnosis of depressive disorder, severe psychiatric disorder, and/or alcohol or drug dependence disorder; 2) non-fluency in mandarin; 3) inability to understand questionnaires or provide consent for themselves. The self-reported questionnaires were distributed in the classrooms with the absence of teachers to protect the privacy of the students and reduce information bias. Based on the matched case-control study formula, the calculated sample size is 27 for each group when investigating the association between parenting style and depressive symptoms, including the parameters in the formula based on previously known parameters: alpha value = 0.05, beta value = 0.20, *P*_0_ = 0.176 (the exposure rate of suffering adverse family care in the control group, OR = 5.41) [27]. The calculated sample size is 78 for each group when investigating the association between *FKBP5* polymorphisms and depressive symptoms, including the parameters in the formula based on previously known evidence: alpha value = 0.05, beta value = 0.20, *P*_0_ = 0.322 (the exposure rate of carrying *FKBP5* rs3800373 allele C in the control group), OR = 1.39 (A vs. C) [28]. In the current nested case-control study, students without depressive symptoms at baseline and follow-up were randomly selected as the control group (*n* = 118), and those with depressive symptoms at baseline and follow-up were treated as the case group (*n* = 120). The case and control groups were matched with age (Fig. 1).

### Measures

#### Depressive symptoms

Depressive symptoms were assessed by the Center for Epidemiologic Studies Depression Scale (CES-D) in Chinese. The Chinese version of this scale has been validated and widely utilized in Chinese studies [29], and shows satisfactory reliability (the total Cronbach’s alpha = 0.88) among Chinese adolescents [30]. The respondents were asked to rate the frequency of 20 symptoms of depression by selecting one of four response options ranging from ‘rarely or none of the time’ to ‘most or all of the time’. The CES-D score ranges from 0 to 60, with higher scores indicating more severe depressive symptomatology [31]. In this study, a score of 28 or higher was applied to identify students with depressive symptoms, and the cutoff score has been used in previous studies among Chinese adolescents [32, 33].

#### Parenting style

Parenting style was measured by asking students their perceptions of their father’s and mother’s parenting style. Based on the Baumrind’s theory, the responses included four types of parenting styles (1 = Authoritative, 2 = Authoritarian or disciplinarian, 3 = Permissive or indulgent, 4 = Neglectful or uninvolved) [34, 35]. Moreover, the explanations of different parenting styles have also been provided as below. Authoritative parenting means high demandingness and responsiveness; although authoritative parents have high expectations for achievement and maturity, they are also warm and responsive. Authoritarian parenting means high demandingness and low responsiveness; high levels of parental control and low levels of responsiveness are the main two characteristics of authoritarian parents. Permissive parenting means low demandingness and high responsiveness; permissive parents set very few rules and boundaries and are reluctant to enforce them. Neglectful parenting means low demandingness and low responsiveness, and neglectful parents are indifferent to their children’s needs or uninvolved in their lives.

### Blood collection and DNA extraction

A peripheral whole blood sample (5 ml) was collected from each student using EDTA anticoagulant tubes at baseline survey (7:00 to 10:00 am), and stored at − 80 °C before use. After the follow-up survey was completed, genomic DNA was extracted from blood samples with the DNA extraction kit (BioTeke Corporation, Beijing, China) according to the manufacturer’s instructions and prepared for genotyping and methylation analysis. DNA concentration was measured at the wavelength of A260 nm by a NanoDrop 2000C spectrophotometer (Thermo Scientific, Waltham, MA, USA).

#### SNP selection and genotyping

Haploview v.4.2 was used to select tagSNPs in linkage disequilibrium (LD) (*r*^2^ > 0.8) with the remaining SNPs at minor allele frequency (MAF) > 0.1 in Han Chinese in Beijing (CHB). Functional SNP (rs3800373 and rs2817035) and SNPs most commonly associated with depressive symptoms or depressive disorder from the literature (rs7748266, rs9470080, rs4713902, rs1360780, rs9380524, rs9394309, rs7757037, rs1043805, rs2766533, rs4713916, rs9296158, rs2817032) [8–11] were prioritized as tagSNPs. In total, 14 SNPs were selected (**Table S1**). Assay Design 4.0 software (Agena Bioscience, Inc., San Diego, CA, USA) was used to design the primers. SNP genotyping was determined following the MassARRAY Nanodispenser (Agena Bioscience) protocols and MassARRAYiPLEX platform (Agena Bioscience) recommended by the manufacturer. None of the selected SNPs had more than 10% genotyping errors or were in severe Hardy-Weinberg disequilibrium (*P* < 0.001). Moreover, LD between the selected polymorphisms of the *FKBP5* gene was examined by pair-wise comparisons of D′ and r^2^ using Haploview v.4.2. One LD block (consisting of the SNPs rs9380524, rs7748266, rs1360780, and rs4713902; r^2^ = 0.08–0.79) were identified and presented in **Fig. S1** (relatively low LD).

#### *FKBP5* promoter methylation analysis

The CpG island in the promoter region of the *FKBP5* gene was selected as the target for methylation analysis. The sequences of CpG island (chr6: 35728998–35,729,370) were determined through the CpG Island Online Prediction website (http://www.ebi.ac.uk/Tools/seqstats/emboss_cpgplot/) based on the CpG island determination criteria (%GC > 50, length > 200 bp, Obs/Exp CpG > 0.6). The methylation of 14 CpG units, encompassing 20 CpG sites, was quantified using the SEQUENOM MassARRAY EpiTYPER platform [36]. Further quality control was performed, including excluding CpG units with less than 80% of available methylation data to ensure that spurious data were not analyzed [37]. Moreover, significantly deviating data points were also excluded [38]. A total of 14 CpG units were ultimately qualified for analysis (**Table S2**).

#### Other information

Demographic information including age, sex (1 = boy, 2 = girl), living arrangement was also collected. Living arrangement was assessed by asking who lived in the student’s primary home (responses were coded as living with both parents = 1, living with a single parent = 2, living with others = 3).

Morning serum cortisol level was also tested. Another 4-mL sample of whole blood was drawn from 7:00 to 10:00 am to obtain serum, and the morning serum total cortisol level was assayed with the competitive chemiluminescent microparticle immunoassay utilizing the Abbott Architect i2000SR system (Abbott Laboratories, Abbott Park, IL).

### Statistical analysis

All statistical analyses were conducted using SPSS (IBM SPSS Statistics for Windows, Version 22.0. Armonk, NY) and R (version 4.1.0, R Core Team, Vienna, Austria). All statistical tests were two-sided, and a *P*-value of < 0.05 was considered statistically significant. Descriptive analyses were used to describe the sample characteristics. Continuous and categorical data were reported in the form of proportions and means (SD). Student t-tests for continuous variables and chi-square tests for categorical variables were conducted to test the differences between the cases and control groups. The distribution of the observed genotype frequencies of *FKBP5* polymorphisms and *FKBP5* methylation levels in the cases and control group was described, and multiple inheritance models were applied to analyze genotype data. The frequency and distribution of haplotypes in cases and controls were analyzed. Conditional logistic regression models were performed to first test the main effects of *FKBP5* polymorphisms and parenting styles on depressive symptoms. The false discovery rate (FDR) was calculated to address the concern of multiple hypothesis testing and potential type I errors. The FDR-adjusted *P* was indicated by “*q*”, and the results were considered as nominally significant when *q* < 0.10 [39]. To investigate interactions between *FKBP5* polymorphisms/methylation status and parenting styles on depressive symptoms, the interaction items between *FKBP5* polymorphisms/methylation status and the parenting style of father/mother were added into the multivariate conditional logistic regression models along with single variables, respectively. To control potential type I errors, the two-sided α-level of 0.05 was corrected into 0.025 (0.05/2 for two-way interaction items of *FKBP5* SNPs × the parenting style of father/mother).

## Results

Table 1 shows the characteristics of the sample. In the students with depressive symptoms (cases), the median age was 13.0 (interquartile range: 13.0 to 15.0) years, the proportion of females was 66.7%, and the proportion of students living with a single parent was 17.5%. In the students without depressive symptoms (controls), the median age was 13.0 (interquartile range: 13.0 to 14.0) years, the proportion of females was 40.7%, and the proportion of students living with a single parent was 8.5%. The differences between the cases and control group in sex and living arrangement distribution were statistically significant (*P* < 0.05). Regarding the parenting style, the proportion of students who reported suffering authoritarian parenting style of the father in cases was 10.8% and in the control group was 3.4%; the proportion of those who reported suffering authoritarian parenting style of the mother in cases was 10.0% and in the control group was 1.7%; the differences of reported parenting style between the cases and control group were statistically significant (*P* < 0.05).

**Table 1:** Sample characteristics between cases and control group

Variable	Non-depressive symptoms group (n, %)	Depressive symptoms group (n, %)	***P*** value*
**Total**	118 (100)	120 (100)	
**Age,** median (interquartile range), year	13.0 (13.0 to 14.0)	13.0 (13.0 to 15.0)	0.118
**Sex**			
Boy	70 (59.3)	40 (33.3)	< 0.001
Girl	48 (40.7)	80 (66.7)	
**Living arrangement**			
Living with both parents	103 (88.0)	86 (71.7)	0.006
Living with a single parent	10 (8.5)	21 (17.5)	
Living with others	4 (3.4)	13 (10.8)	
Missing data	1		
**Morning serum total cortisol**, median (interquartile range), nmol/L	224.8 (155 to 318)	197.6 (147.9 to 290.8)	0.152
**Parenting style of the father**			
Permissive	81 (68.6)	67 (55.8)	< 0.001
Authoritative	32 (27.1)	25 (20.8)	
Authoritarian	4 (3.4)	13 (10.8)	
Neglectful	1 (0.8)	15 (12.5)	
**Parenting style of the mother**			
Permissive	100 (84.7)	78 (65.0)	0.001
Authoritative	16 (13.6)	24 (20.0)	
Authoritarian	2 (1.7)	12 (10.0)	
Neglectful	0	6 (5.0)	

*: The chi-square test was used for categorical variables, and the Wilcoxon rank test was used for age and morning serum total cortisol data

The genotype frequency distributions of the *FKBP5* polymorphisms in the cases and control group are shown in **Table S1**. As shown in Table 2, without adjusting for other variables, only rs7757037, rs2817032, and rs2817035 were associated with depressive symptoms under the codominant model and dominant model (even after further correction for multiple testing, *q* < 0.10). After adjusting for age, gender, living arrangement, and morning cortisol level, a significant association between rs7757037 and depressive symptoms was found in the codominant model (AG vs. GG; adjusted odds ratio [AOR] = 2.56, 95% CI = 1.13–5.78, *q* < 0.10) and the dominant model (AA+AG vs. GG; AOR = 2.38, 95% CI = 1.11–5.12, *q* < 0.10); rs2817032 polymorphism was associated with depressive symptoms in the codominant model (TT vs. CC; AOR = 3.63, 95% CI = 1.28–10.25, *q* < 0.10 & TC vs. CC; AOR = 4.01, 95% CI = 1.35–11.89, *q* < 0.10) and dominant model (TT + TC vs. CC; AOR = 3.76, 95% CI = 1.36–10.38, *q* < 0.10); rs2817035 was also associated with depressive symptoms in the codominant model (GG vs. AA; AOR = 2.93, 95% CI = 1.08–7.96, *q* < 0.10) and dominant model (GG + GA vs. AA, AOR = 2.78, 95% CI = 1.05–7.36, *q* < 0.10). However, after further adjusting for the father’s or mother’s parenting style, there are no significant associations of rs7757037, rs2817032, and rs2817035 with depressive symptoms (*P* > 0.05; after further correction for multiple testing, *q* > 0.10). **Table S2** shows that the haplotype frequencies were not significantly different between the cases and the controls (*P* > 0.05).

**Table 2:** Main effects of the *FKBP5* polymorphisms on depressive symptoms

Variable		Model 1			Model 2			Model 3			Model 4		
		OR (95% CI)	***P*** value	***q*** value	Adjusted OR (95% CI)	***P*** value	***q*** value	Adjusted OR (95% CI)	***P*** value	***q*** value	Adjusted OR (95% CI)	***P*** value	***q*** value
rs7757037^*^													
Codominant model	AA vs. GG	2.01 (0.94–4.34)	0.074	0.111	2.17 (0.93–5.01)	0.072	0.108	2.02 (0.86–4.78)	0.109	0.163	1.98 (0.85–4.64)	0.116	0.174
	AG vs. GG	2.35 (1.13–4.92)	0.023	0.075**	2.56 (1.13–5.78)	0.024	0.062**	2.34 (1.02–5.36)	0.044	0.115	2.02 (0.89–4.63)	0.095	0.174
Dominant model	AA+AG vs. GG	2.20 (1.10–4.39)	0.025	0.075**	2.38 (1.11–5.12)	0.026	0.062**	2.20 (1.01–4.78)	0.048	0.115	2.00 (0.93–4.33)	0.077	0.174
Recessive model	AA vs. AG + GG	1.09 (0.63–1.89)	0.748	0.748	1.10 (0.61–2.00)	0.744	0.744	1.10 (0.60–2.02)	0.762	0.762	1.21 (0.66–2.23)	0.543	0.543
rs2817032^*^													
Codominant model	TT vs. CC	3.01 (1.18–7.70)	0.022	0.075**	3.63 (1.28–10.25)	0.015	0.060**	3.12 (1.09–8.89)	0.034	0.115	2.90 (1.02–8.27)	0.046	0.174
	TC vs. CC	2.77 (1.04–7.35)	0.041	0.086**	4.01 (1.35–11.89)	0.012	0.060**	3.16 (1.05–9.55)	0.041	0.115	3.09 (1.03–9.24)	0.044	0.174
Dominant model	TT + TC vs. CC	2.91 (1.17–7.27)	0.022	0.075**	3.76 (1.36–10.38)	0.011	0.060**	3.13 (1.12–8.74)	0.029	0.115	2.97 (1.07–8.25)	0.037	0.174
Recessive model	TT vs. TC + CC	1.36 (0.81–2.28)	0.243	0.265	1.24 (0.71–2.15)	0.456	0.497	1.29 (0.73–2.29)	0.385	0.420	1.22 (0.69–2.16)	0.501	0.543
rs2817035^*^													
Codominant model	GG vs. AA	2.55 (1.03–6.32)	0.043	0.086**	2.93 (1.08–7.96)	0.035	0.069**	2.49 (0.90–6.86)	0.079	0.158	2.37 (0.86–6.54)	0.096	0.174
	GA vs. AA	2.07 (0.80–5.37)	0.135	0.180	2.52 (0.88–7.20)	0.085	0.113	2.05 (0.70–5.99)	0.188	0.251	2.03 (0.70–5.90)	0.194	0.259
Dominant model	GG + GA vs. AA	2.36 (0.98–5.72)	0.056	0.096**	2.78 (1.05–7.36)	0.040	0.069**	2.32 (0.86–6.25)	0.095	0.163	2.24 (0.84–6.02)	0.110	0.174
Recessive model	GG vs. GA + AA	1.47 (0.87–2.47)	0.151	0.181	1.45 (0.83–2.54)	0.197	0.236	1.44 (0.81–2.57)	0.219	0.263	1.38 (0.77–2.46)	0.275	0.330
**Parenting style of the father**													
Authoritarian		1.00 (reference)			1.00 (reference)								
Permissive		0.26 (0.08–0.82)	0.021	NA	0.19 (0.06–0.66)	0.009	NA	NA	NA	NA	NA	NA	NA
Authoritative		0.24 (0.07–0.83)	0.024	NA	0.22 (0.06–0.82)	0.023	NA	NA	NA	NA	NA	NA	NA
Neglectful		4.62 (0.46–46.67)	0.195	NA	3.15 (0.29–34.59)	0.348	NA	NA	NA	NA	NA	NA	NA
**Parenting style of the mother**													
Authoritarian		1.00 (reference)			1.00 (reference)								
Permissive		0.13 (0.03–0.60)	0.009	NA	0.11 (0.02–0.53)	0.006	NA	NA	NA	NA	NA	NA	NA
Authoritative		0.25 (0.05–1.27)	0.095	NA	0.22 (0.04–1.21)	0.081	NA	NA	NA	NA	NA	NA	NA
Neglectful		NA	NA	NA	NA	NA	NA	NA	NA	NA	NA	NA	NA

Abbreviations: OR, odds ratio; 95% CI, 95% confidence interval; NA, not applicable or not available

*: Only the *FKBP5* polymorphisms observed with a significant association of depressive symptoms in the unadjusted model were reported here

Model 1: unadjusted models

Model 2: adjusted for age, gender, living arrangement, and morning cortisol level

Model 3: adjusted for age, gender, living arrangement, morning cortisol level, and parenting style of the father

Model 4: adjusted for age, gender, living arrangement, morning cortisol level, and parenting style of the mother

**: “*q* value” indicates the FDR-adjusted *P* value, and the results were considered as nominally significant when *q* < 0.10

Table 3 depicts the interaction effects between *FKBP5* polymorphisms and parenting style on depressive symptoms. After adjusting for age, gender, living arrangement, and morning cortisol level, significant interactions between rs7757037 and the father’s parenting style were found in the codominant model (AG vs. GG; *P* = 0.043) and dominant model (AA+AG vs. GG; *P* = 0.043). However, the gene-environment interactions were not significant after correcting for multiple testing.

**Table 3:** Interaction effects between *FKBP5* polymorphisms and parenting style on depressive symptoms

Interaction item		Model 1		Model 2	
		OR (95% CI)	***P*** value	Adjusted OR (95% CI)	***P*** value
**rs7757037**					
rs7757037 × parenting style of the father					
Codominant model	AA vs. GG	5.35 (1.15–24.8)	0.032	4.62 (0.90–23.85)	0.067
	AG vs. GG	6.04 (1.36–26.94)	0.018^*^	5.13 (1.05–25.06)	0.043
Dominant model	AA+AG vs. GG	5.78 (1.34–24.96)	0.019^*^	4.94 (1.05–23.27)	0.043
Recessive model	AA vs. AG + GG	1.05 (0.51–2.18)	0.890	1.06 (0.47–2.40)	0.889
rs7757037 × parenting style of the mother					
Codominant model	AA vs. GG	0.70 (0.11–4.43)	0.702	0.22 (0.03–1.65)	0.139
	AG vs. GG	0.72 (0.12–4.21)	0.715	0.26 (0.04–1.75)	0.165
Dominant model	AA+AG vs. GG	0.71 (0.13–4.02)	0.703	0.25 (0.04–1.60)	0.142
Recessive model	AA vs. AG + GG	0.87 (0.32–2.36)	0.783	0.67 (0.22–2.05)	0.478
**rs2817032**					
rs2817032 × parenting style of the father					
Codominant model	TT vs. CC	NA	NA	NA	NA
	TC vs. CC	NA	NA	NA	NA
Dominant model	TT + TC vs. CC	NA	NA	NA	NA
Recessive model	TT vs. TC + CC	1.10 (0.56–2.15)	0.784	1.04 (0.49–2.21)	0.912
rs2817032 × parenting style of the mother					
Codominant model	TT vs. CC	NA	NA	NA	NA
	TC vs. CC	NA	NA	NA	NA
Dominant model	TT + TC vs. CC	NA	NA	NA	NA
Recessive model	TT vs. TC + CC	0.98 (0.37–2.57)	0.969	0.61 (0.21–1.79)	0.372
**rs2817035**					
rs2817035 × parenting style of the father					
Codominant model	GG vs. AA	7.60 (0.71–81.21)	0.094	8.30 (0.70–97.85)	0.093
	GA vs. AA	7.50 (0.70–80.67)	0.097	7.95 (0.66–95.40)	0.102
Dominant model	GG + GA vs. AA	7.43 (0.71–77.64)	0.094	8.14 (0.71–93.65)	0.093
Recessive model	GG vs. GA + AA	1.10 (0.56–2.14)	0.787	1.15 (0.54–2.45)	0.714
rs2817035 × parenting style of the mother					
Codominant model	GG vs. AA	NA	NA	NA	NA
	GA vs. AA	NA	NA	NA	NA
Dominant model	GG + GA vs. AA	NA	NA	NA	NA
Recessive model	GG vs. GA + AA	1.19 (0.46–3.05)	0.722	0.90 (0.32–2.58)	0.850

Abbreviations: OR, odds ratio; 95% CI, 95% confidence interval; NA, not applicable or not available

Model 1: unadjusted models

Model 2: adjusted for age, gender, living arrangement, and morning cortisol level

*: Gene-environment interactions, which remained significant after correcting for multiple testing

As shown in **Table S3**, we observed no significant differences in the methylation levels of the selected *FKBP5* CpG sites between the cases and the control group (all *P* > 0.05). Additionally, there were no significant interactions between *FKBP5* gene methylation status and parenting styles on depressive symptoms observed in this study (all *P* > 0.05, Table 4).

**Table 4:** Interaction effects between *FKBP5* gene methylation status and parenting style on depressive symptoms

Interaction	***P*** value^*^
**Parenting style of the father×**	
*FKBP5*–12 CpG 1	0.653
*FKBP5*–12 CpG 2	0.974
*FKBP5*–12 CpG 3	0.414
*FKBP5*–12 CpG 4	0.761
*FKBP5*–12 CpG 5.6.7	0.951
*FKBP5*–12 CpG 8	0.630
*FKBP5*–12 CpG 9	0.887
*FKBP5*–12 CpG 10.11	0.942
*FKBP5*–12 CpG 12	0.232
*FKBP5*–12 CpG 13	0.463
*FKBP5*–12 CpG 14	0.599
*FKBP5*–12 CpG 15	0.491
*FKBP5*–12 CpG 17.18.19	0.505
*FKBP5*–12 CpG 20	0.761
**Parenting style of the mother×**	
*FKBP5*–12 CpG 1	0.951
*FKBP5*–12 CpG 2	0.661
*FKBP5*–12 CpG 3	0.575
*FKBP5*–12 CpG 4	0.211
*FKBP5*–12 CpG 5.6.7	0.230
*FKBP5*–12 CpG 8	0.217
*FKBP5*–12 CpG 9	0.852
*FKBP5*–12 CpG 10.11	0.818
*FKBP5*–12 CpG 12	0.063
*FKBP5*–12 CpG 13	0.205
*FKBP5*–12 CpG 14	0.486
*FKBP5*–12 CpG 15	0.498
*FKBP5*–12 CpG 17.18.19	0.766
*FKBP5*–12 CpG 20	0.211

*: The interaction items between *FKBP5* methylation status and the parenting style of father/mother were added into the multivariate conditional logistic regression models along with single variables, respectively. Considering the 95% confidence intervals of the odds ratios were too wide, only *P* values were reported

## Discussion

Considering that the stress-related gene *FKBP5* may play a prominent role in depressive symptoms, this study investigated *FKBP5* polymorphisms and methylations as potential candidates for gene-environment influences on depressive symptoms in an adolescent sample. Our findings suggested that among the selected 14 SNPs, only *FKBP5* rs7757037, rs2817032, and rs2817035 were associated with the increased risk of depressive symptoms in the codominant model and dominant model with and without adjusting for sociodemographic characteristics. Similarly, Piechaczek et al. reported that no main genetic effects of the five SNPs (rs3800373, rs9296158, rs1360780, rs9470080, and rs4713916) on depression were found [13]; Lou et al. reported that rs7757037 of *FKBP5* was associated with depression in Chinese systemic lupus erythematosus patients [40] in dominant model. However, this finding was inconsistent with a study among patients with coronary artery disease, indicating rs2817032 was not associated with depressive symptoms among those patients [41]. The diversity of populations, which might result in various gene sensitivity, may explain the discrepancy of genotype models, while this study focused on Chinese adolescents.

Previous evidence has suggested that parenting style was one of the most significant environmental stressors influencing their child’s growth [22]. Consistent with prior studies [24, 42], the protective role of the father’s permissive and authoritative parenting style on the development of depressive symptoms among Chinese adolescents was observed. Moreover, this study found that the significant genetic main effects of *FKBP5* rs7757037, rs2817032, and rs2817035 were not significant after adjusting for the parenting style of the father or mother, respectively. These findings were in line with most prior studies, which demonstrated no main genetic effects predicting case-control status after adjusting for other variables [12, 13, 15]; indicating that genetic factors may have to interact with environmental stressors to elicit depressive symptoms [19].

Considering that the *FKBP5* gene plays a vital role in regulating the HPA-axis and is implicated in depressive symptoms [18], it seems appropriate to study the effect of the *FKBP5* gene in the context of parenting style as an environmental stressor. Extending previous evidence, a novel aspect of this study was that the influences of the parenting style of father/mother (reflecting more stable living background) and their interactions with *FKBP5* polymorphisms on adolescent depressive symptoms were explored. In contrast, much of the previous literature on the gene-environment interactions at the *FKBP5* locus in the context of depressive symptoms mainly focused on adverse or traumatic life events [15, 20]. In this study, without adjusting for sociodemographic variables, significant interactions between *FKBP5* rs7757037 and the father’s parenting style were first observed in the codominant and dominant model, even correcting for multiple testing. These results might be explained by the diathesis-stress model of depressive symptoms [43], which indicated that *FKBP5* rs7757037 carriers might exhibit a heightened HPA response activity and be more likely to be implicated in the risk for depressive symptoms when experiencing negative parenting styles. However, based on multiple testing corrections, these interaction effects did not significantly predict depressive symptoms after adjusting for sociodemographic variables. These interactions reached nominal significance, and the relatively small sample size in the present study needs to be considered. It would be significant to follow up on this finding in future studies using larger sample sizes. Besides, this finding may also reflect that a single environmental stressor (i.e., parenting style in this study) may not be potent enough to elicit depressive symptoms in adolescence, and other sociodemographic stressors may influence the effects of the single one environmental stress.

Additionally, *FKBP5* epigenetic changes induced by environmental stressors may also be associated with the risk of depressive symptoms [44]. Considering parenting style may affect the developing brain through leading to changes in methylation levels of *FKBP5*, this study also compared the difference of *FKBP5* methylation levels between students with and without depressive symptoms, and investigated the interactions between *FKBP5* methylation status and parenting style. However, no significant findings were observed. Similarly, Klinger-König et al. reported there were no significant effects of *FKBP5* methylation or the interaction between *FKBP5* methylation and childhood maltreatment on depressive symptoms [45]. Höhne et al. showed that no significant difference of *FKBP5* DNA methylation in intron 7 between subjects with a lifetime history of depression and healthy controls was observed [46]. Bustamante et al. also reportedly did not observe any association of *FKBP5* methylation levels in intron 7 or intron 2 with depressive symptoms [47]. These results might be related to the complex relationship between parenting style, *FKBP5* methylation, and depressive symptoms, highlighting that multiple factors may contribute to the development of depressive symptoms following exposure to different parenting styles. Moreover, parenting style exposures have been shown to be long-lasting, and they may not only influence depressive symptoms through the *FKBP5* methylation pathway.

To our knowledge, the present study is the first nested case-control study to explore the associations of *FKBP5* genetic and epigenetic variation with depressive symptoms among Chinese adolescents in the context of parenting style. However, several limitations should be noted. First, only the *FKBP5* gene was examined in this study by a hypothesis-driven approach. Considering this study only focused on the effects of the *FKBP5* gene, other potential genes with implications in depressive symptoms (e.g., *BDNF* or *NR3C1* gene) were not considered, and we would like to explore the effects of other genes on depressive symptoms in our future study. Second, although parenting style and depressive symptoms were measured by self-reported, which may lead to self-report bias, self-reports remain a common and accepted method. Third, considering the questionnaire length, parenting style was not assessed by the scales like the Egna Minnen Beträffande Uppfostran (EMBU) in this study, then the lack of evaluating psychometric properties for the parenting style measure in this study may be a limitation. Fourth, although previous studies have used a similar sample size to explore the associations between the *FKBP5* gene and depressive symptoms or depression [45, 47], the sample size in the present study is relatively small, which may imply insufficient statistical power these findings.

## Conclusions

This study suggests significant relationships of *FKBP5* rs7757037, rs2817032, and rs2817035 with depressive symptoms without adjusting for parenting style and observes a nominally significant interaction between *FKBP5* rs7757037 and parenting style of the father on depressive symptoms. However, there was no significant association between *FKBP5* CpG methylation status and the interactions between *FKBP5* CpG methylation and parenting style with depressive symptoms. Therefore, this work suggests that parenting style, almost experiencing by each adolescent, can be targeted in prevention strategies, and a particular focus should be placed on adolescents who suffer negative parenting styles. Moreover, this study also indicates that *FKBP5* variation, not DNA methylation, may be more sensitive in moderating the effects of parenting style stressors on depressive symptoms, especially for the negative parenting style of the father, even though the evidence under the mechanism is deficient now. Further studies to investigate the underlying mechanism are warranted.

## Supplementary Information

**Additional file 1 Table S1.** Genotype frequency distribution of the FKBP5 polymorphisms between students with and without depressive symptoms. **Fig. S1.** The linkage disequilibrium plots for the SNPs in FKBP5. D′ is a measure of linkage disequilibrium between two genetic markers. A value of D′ = 1 (complete LD) indicates that SNPs have not been separated by recombination, while values of D′ < 1 (incomplete LD) indicate that the ancestral LD was disrupted during the history of the population. The r^2^ is a measure of linkage disequilibrium between two genetic markers. For SNPs that have not been separated by recombination or have the same allele frequencies (perfect LD), *r*^2^ = 1. **Table S2.** Haplotypes of FKBP5 gene and depressive symptoms. **Table S3.** Methylation levels at CpG sites in the promoter region of FKBP5 between students with and without depressive symptoms

## Data Availability

The datasets used and/or analyzed during the current study are available from the corresponding author on reasonable request.
