# Supplementary material for: Associations of FKBP5 polymorphisms and methylation and parenting style with depressive symptoms among Chinese adolescents
Source: BMC Psychiatry. 2021 Nov 9;21:552. doi: 10.1186/s12888-021-03576-6 (PMC8579536; doi:10.1186/s12888-021-03576-6)
Supplement: Supplementary file 1 — Additional file 1 Table S1. Genotype frequency distribution of the FKBP5 polymorphisms between students with and without depressive symptoms. Fig. S1. The linkage disequilibrium plots for the SNPs in FKBP5. D′ is a measure of linkage disequilibrium between two genetic markers. A value of D′ = 1 (complete LD) indicates that SNPs have not been separated by recombination, while values of D′ < 1 (incomplete LD) indicate that the ancestral LD was disrupted during the history of the population. The r2 is a measure of linkage disequilibrium between two genetic markers. For SNPs that have not been separated by recombination or have the same allele frequencies (perfect LD), r2 = 1. Table S2. Haplotypes of FKBP5 gene and depressive symptoms. Table S3. Methylation levels at CpG sites in the promoter region of FKBP5 between students with and without depressive symptoms [file 12888_2021_3576_MOESM1_ESM.docx]

| **Table S1.** **Genotype frequency distribution of the *FKBP5* polymorphisms between students with and without depressive symptoms** | | | | |
| --- | --- | --- | --- | --- |
| **Variable** | **Depressive symptoms**  **group (n=120)** | | **Non-depressive symptoms**  **group (n=118)** | ***P* value*** |
| rs7748266 | |  |  |  |
| CC | | 75 (63.6) | 65 (56.0) | 0.324 |
| CT | | 34 (28.8) | 36 (31.0) |  |
| TT | | 9 (7.6) | 15 (12.9) |  |
| rs9470080 | |  |  |  |
| CC | | 57 (48.7) | 47 (40.5) | 0.129 |
| CT | | 49 (41.9) | 48 (41.4) |  |
| TT | | 11 (9.4) | 21 (18.1) |  |
| rs4713902 | |  |  |  |
| TT | | 68 (58.6) | 74 (64.3) | 0.645 |
| TC | | 45 (38.8) | 39 (33.9) |  |
| CC | | 3 (2.6) | 2 (1.7) |  |
| rs1360780 | |  |  |  |
| CC | | 68 (58.1) | 59 (52.2) | 0.456 |
| CT | | 39 (33.3) | 39 (34.5) |  |
| TT | | 10 (8.5) | 15 (13.3) |  |
| rs9380524 | |  |  |  |
| CC | | 40 (34.2) | 51 (44.3) | 0.178 |
| CA | | 64 (54.7) | 49 (42.9) |  |
| AA | | 13 (11.1) | 15 (13.0) |  |
| rs9394309 | |  |  |  |
| AA | | 74 (62.7) | 63 (54.3) | 0.392 |
| AG | | 34 (28.8) | 39 (33.6) |  |
| GG | | 10 (8.5) | 14 (12.1) |  |
| rs7757037 | |  |  |  |
| AA | | 41 (36.0) | 38 (33.9) | 0.067 |
| AG | | 58 (50.9) | 46 (41.1) |  |
| GG | | 15 (13.2) | 28 (25.0) |  |
| rs1043805 | |  |  |  |
| AA | | 74 (63.8) | 68 (58.6) | 0.721 |
| AT | | 35 (30.2) | 40 (34.5) |  |
| TT | | 7 (6.0) | 8 (6.9) |  |
| rs2766533 | |  |  |  |
| GG | | 59 (50.0) | 49 (42.2) | 0.141 |
| GA | | 50 (42.4) | 49 (42.2) |  |
| AA | | 9 (7.6) | 18 (15.5) |  |
| rs3800373 | |  |  |  |
| AA | | 67 (57.8) | 56 (49.6) | 0.250 |
| AC | | 39 (33.6) | 40 (35.4) |  |
| CC | | 10 (8.6) | 17 (15.0) |  |
| rs4713916 | |  |  |  |
| AA | | 75 (63.6) | 63 (54.3) | 0.237 |
| AG | | 35 (29.7) | 39 (33.6) |  |
| GG | | 8 (6.8) | 14 (12.1) |  |
| rs9296158 | |  |  |  |
| AA | | 55 (49.5) | 47 (41.2) | 0.227 |
| AG | | 46 (41.4) | 49 (43.0) |  |
| GG | | 10 (9.0) | 18 (15.8) |  |
| rs2817032 | |  |  |  |
| **Table S1. Genotype frequency distribution of the *FKBP5* SNPs** **between students with and without depressive symptoms (continued)** | | | | |
| TT | | 69 (58.5) | 59 (50.9) | 0.057 |
| TC | | 42 (35.6) | 39 (33.6) |  |
| CC | | 7 (5.9) | 18 (15.5) |  |
| rs2817035 | |  |  |  |
| GG | | 72 (61.5) | 60 (52.2) | 0.115 |
| GA | | 37 (31.6) | 38 (33.0) |  |
| AA | | 8 (6.8) | 17 (14.8) |  |

*: The chi-square test was used for categorical variables.


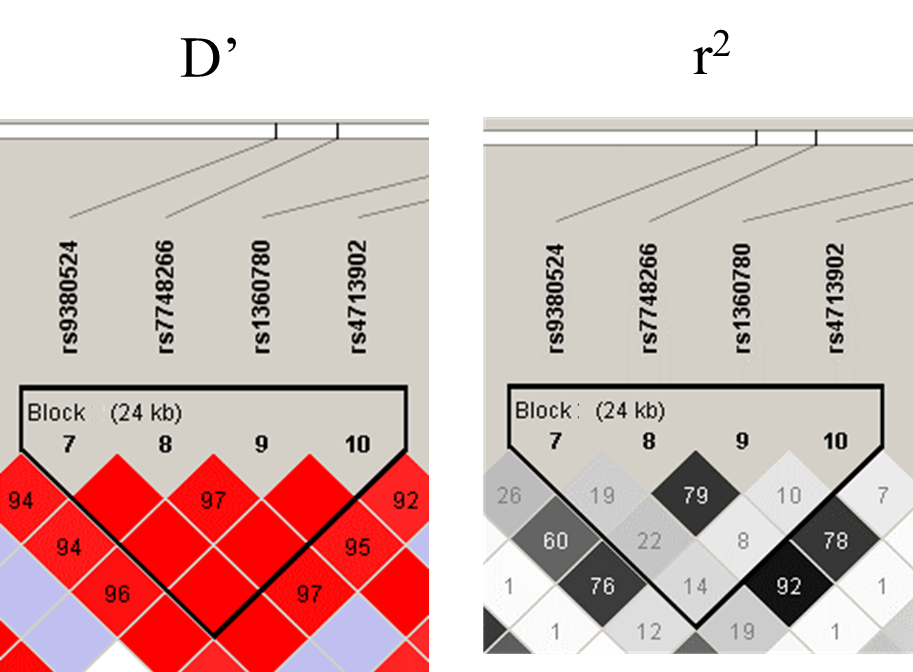


**Figure S1.** The linkage disequilibrium plots for the SNPs in FKBP5. D’ is a measure of linkage disequilibrium between two genetic markers. A value of D’= 1 (complete LD) indicates that SNPs have not been separated by recombination, while values of D’< 1 (incomplete LD) indicate that the ancestral LD was disrupted during the history of the population. The r^2^ is a measure of linkage disequilibrium between two genetic markers. For SNPs that have not been separated by recombination or have the same allele frequencies (perfect LD), r^2^ = 1.

| **Table S2. Haplotypes of FKBP5 gene and depressive symptoms** | | | | |
| --- | --- | --- | --- | --- |
| **Hyplotype** | **Frequency** | **Case: control**  **frequencies** | Chi-square | ***P* value** |
| **Block** |  |  |  |  |
| ACCT | 0.363 | 0.344, 0.381 | 0.721 | 0.396 |
| CTTT | 0.248 | 0.280, 0.216 | 2.541 | 0.111 |
| CCCC | 0.205 | 0.188, 0.221 | 0.793 | 0.373 |
| CCCT | 0.141 | 0.144, 0.138 | 0.035 | 0.852 |
| CCTT | 0.04 | 0.040, 0.039 | 0.002 | 0.964 |

| **Table S3.** **Methylation levels at CpG sites in the promoter region of *FKBP5* between students with and without depressive symptoms** | | | |
| --- | --- | --- | --- |
| **CPG unit** | **Non-depressive symptoms group (n=118)** | **Depressive symptoms group (n=120)** | ***P* value*** |
| *FKBP5*-12 CpG 1 | 0.73 (1.85) | 0.77 (1.46) | 0.861 |
| *FKBP5*-12 CpG 2 | 2.92 (2.40) | 3.06 (2.36) | 0.644 |
| *FKBP5*-12 CpG 3 | 3.13 (3.58) | 2.34 (2.89) | 0.064 |
| *FKBP5*-12 CpG 4 | 2.48 (4.95) | 3.33 (5.59) | 0.217 |
| *FKBP5*-12 CpG 5.6.7 | 40.97 (15.69) | 40.19 (16.59) | 0.713 |
| *FKBP5*-12 CpG 8 | 5.72 (5.81) | 5.64 (10.12) | 0.940 |
| *FKBP5*-12 CpG 9 | 5.46 (5.41) | 4.79 (4.72) | 0.310 |
| *FKBP5*-12 CpG 10.11 | 3.74 (8.96) | 3.87 (9.01) | 0.910 |
| *FKBP5*-12 CpG 12 | 14.67 (14.01) | 12.85 (13.22) | 0.304 |
| *FKBP5*-12 CpG 13 | 53.96 (21.14) | 50.16 (23.14) | 0.187 |
| *FKBP5*-12 CpG 14 | 2.84 (2.42) | 3.37 (2.92) | 0.128 |
| *FKBP5*-12 CpG 15 | 2.63 (3.47) | 2.42 (3.25) | 0.617 |
| *FKBP5*-12 CpG 17.18.19 | 14.15 (9.27) | 14.27 (10.41) | 0.919 |
| *FKBP5*-12 CpG 20 | 2.48 (4.95) | 3.33 (5.59) | 0.217 |

Note: *FKBP5*-12 represents DNA fragment position corresponding to primer #12.

*: Data were presented as Mean ± SD, and student’s t-tests were used to estimate the differences between students with and without depressive symptoms.
